# Influence of slope steepness, foot position and turn phase on plantar pressure distribution during giant slalom alpine ski racing

**DOI:** 10.1371/journal.pone.0176975

**Published:** 2017-05-04

**Authors:** Thomas Falda-Buscaiot, Frédérique Hintzy, Patrice Rougier, Patrick Lacouture, Nicolas Coulmy

**Affiliations:** 1 Laboratoire Interuniversitaire de biologie de la motricité, Université Savoie Mont Blanc, Chambéry, France; 2 Cluster Sporaltec, Saint Etienne, France; 3 Laboratoire RoBioSS (UPR 3346), institut Pprime, Université de Poitiers, CNRS, Poitiers, France; 4 Département Sportif et Scientifique, Fédération Française de ski, Annecy, France; Universite de Nantes, FRANCE

## Abstract

The purpose of this study was to investigate the evolution of ground reaction force during alpine skiing turns. Specifically, this study investigated how turn phases and slope steepness affected the whole foot normal GRF pattern while performing giant slalom turns in a race-like setting. Moreover, the outside foot was divided into different plantar regions to see whether those parameters affected the plantar pressure distribution. Eleven skiers performed one giant slalom course at race intensity. Runs were recorded synchronously using a video camera in the frontal plane and pressure insoles under both feet’s plantar surface. Turns were divided according to kinematic criteria into four consecutive phases: initiation, steering1, steering2 and completion; both steering phases being separated by the gate passage. Component of the averaged Ground Reaction Force normal to the ski’s surface($\bar{nGRF}$, /BW), and Pressure Time Integral relative to the entire foot surface (relPTI, %) parameters were calculated for each turn phases based on plantar pressure data. Results indicated that $\bar{nGRF}$ under the total foot surface differed significantly depending on the slope (higher in steep sections vs. flat sections), and the turn phase (higher during steering2 vs. three other phases), although such modifications were observable only on the outside foot. Moreover, $\bar{nGRF}$ under the outside foot was significantly greater than under the inside foot.RelPTI under different foot regions of the outside foot revealed a global shift from forefoot loading during initiation phase, toward heel loading during steering2 phase, but this was dependent on the slope studied. These results suggest a differentiated role played by each foot in alpine skiing turns: the outside foot has an active role in the turning process, while the inside foot may only play a role in stability.

## Introduction

Pressure insoles are a useful measurement system to assess kinetic parameters during posture, gait or dynamic activities in field situations, since they have a minimal influence on the subject’s skill (e.g. [1,2]). Indeed, pressure insoles are placed inside the shoe and are thin and light. Therefore, various investigators have recently used them during alpine skiing to better understand the relationship between kinematic and kinetic parameters, and their effects on performance, training and injury [3–8]. This measurement system converts raw pressure signals into the magnitude and the distribution of one directional (compressive) force (i.e., the component of the Ground Reaction Force normal to the ski’s surface, nGRF), beneath the entire plantar surface. It is further possible to divide the plantar surface into specific anatomical regions. The determination of the point of application of the reaction force (i.e., center of pressure, CoP) is therefore possible, a parameter that has been shown to be able to characterize learning processes in alpine skiing [9]. However, several limitations should be pointed out. The compressive force is underestimated from 21% to 54% compared to a force platform [6], and this underestimation varies depending on the phase of the turn, the skier’s skill level, the pitch of the slope and the skiing mode [7]. It has been stated this underestimation originates from a significant part of the force actually being transferred through the ski boot’s cuff [6,7,10]. As a result, the CoP trajectory also tends to be underestimated along both the anterior-posterior (A-P) and medial-lateral (M-L) axes compared to force platforms [8]. Nonetheless, analysis of the Ground Reaction Force (GRF) and CoP measured with pressure insoles may provide useful information on alpine skiing biomechanics and kinaesthetic feelings of the skiers.

Previous studies have mainly investigated the nGRF applied throughout a turn. This parameter was calculated from both pressure and surface area data, relative to the body weight (BW). nGRF ranged from 0.2 times BW on the inside foot [7], to 1.2 times BW [7] or 2 times BW [5] on the outside foot during slalom or giant slalom turns. This wide range of data was partly explained by the foot studied (higher nGRF on the outside vs. inside foot; [5] and the phase of the turn (higher nGRF at the end of the steering phase; [11,12]). However, nGRF is also likely to be influenced by other factors such as the skier’s skill [13], slope steepness [14], course setting [15], skiing mode [7], or skier’s body position [16]. To the best of our knowledge, no study has investigated these plausible factors together. Although the above-mentioned studies differed in course setting, population studied or snow quality, a consensus seemed to be established in terms of a global ground reaction peak during a turn at about 2500 N [17].

The CoP trajectory during alpine skiing turns has also been investigated with pressure insoles. It has been reported that CoP shifts backward from the beginning of a turn until the steering phase [3,4], although no data have been presented to support this finding. Nakazato et al. [8] also measured a backward shift of the CoP during a turn. Its amplitude may vary according to experimental conditions.

Another use of pressure insole measurements is their distribution under the entire surface of the plantar sole. Interestingly, by partitioning the total foot surface into different anatomical regions, Lamontagne [5] showed that the highest pressures were found under the heel, the first metatarsal and the hallux of the outside foot, with values up to 16 N.cm^2^ during slalom and giant slalom turns. Such values were found when averaged over an entire foot sole region. When using individualized sensors, values of up to 30 N.cm^2^ were found [3,4]. However, no information was provided concerning how this distribution evolved throughout the turn, whereas a modification could be hypothesized depending on turn completion.

In conclusion, these studies have highlighted a major contribution of different factors to the nGRF applied throughout a turn, such as the foot’s position during a turn (inside vs. outside), the CoP A-P displacement, or precise loading of different foot sole regions. Unfortunately, these results have been studied separately. Since these factors are likely related, their relative contribution to nGRF could be modified when combined effects are analyzed. Although it was not their main purpose, Nakazato et al. showed that skiing mode, pitch of the slope, phase of the turn and skier skill were factors that likely affect the nGRF measured by pressure insoles [7].

The aims of the present study were to investigate i) the influence of turn phases and modifications in slope steepness on the whole foot normal GRF pattern while performing giant slalom turns in a race-like setting, and ii) how these parameters affect plantar pressure distribution under the feet.

## Materials and methods

### Subjects

Eleven young experienced alpine skiers participated in the study (seven boys and four girls). Their mean age, mass and height were 17.5 ± 4.3 years, 59.8 ± 10.6 kg and 170.3 ± 8.2 cm, respectively. All skiers were competing at a regional level at the time of the study (national ranking: 105 ± 37 points). Prior to the experiment, all the skiers were informed of the purpose, risks, and benefits of the experiment. The study was approved by the ethics committee of the University Savoie Mont-Blanc and written informed consent was obtained from each subject prior to the study. For subjects who were minor at the time of the study, additional written informed consent was obtained from their parents. The study was conducted according to the 1964 declaration of Helsinki. The subjects used their own ski racing equipment (race suit, skis, boots, etc.).

### Experimental protocol

The test took place on a closed ski slope in the St Gervais (France) ski resort on two consecutive days. The slope was homologated by the French Ski Federation for race use. The slope had sections of different pitch ranging from 28° (top part of the slope) to 11° (middle part), and no side hill sections. All measurements were taken under optimal conditions: good and consistent weather conditions, groomed and hard snow, and the course was side-slipped after each run. A giant slalom course was set up by a professional, certified trainer, who also used a laser to measure distances between gates while setting them. The complete course had a vertical drop of 250 m and 30 gates. The mean linear distance and lateral offset between gates were 25 m and 9 m in the steep slope condition, and 31 and 4 m for the flat slope condition. Laser measurement was used while setting the gates. Linear distance and lateral offset were very consistent between gates with similar steepness to ensure valid averaging of subsequent turns.

The experiment consisted of one run performed by each subject, who were told to complete it at race intensity. The run was timed using regular photocells (HL2, Tag Heuer, La Chaux de Fond, Switzerland). Free skiing warm-up and inspection of the course were undertaken prior to the run.

Two consecutive turns at the top part of the course were analyzed, providing data for the “steep” slope condition (28°), as were six consecutive turns in the middle part of the course, providing data for the “flat” slope condition (11°). All of the analyzed gates were preceded and followed by another gate in the same slope condition (steep or flat). Consecutive turns were analyzed as it has been shown no differences existed between plantar pressure applied under the right and left feet [5]. The skier’s feet were analyzed according to the foot position during a turn: either inside (e.g., the left foot in a left-side turn) or outside (e.g., the right foot in a left-side turn).

### Instrumentation and data processing

All runs were recorded with a fixed digital camera (50Hz) placed in the frontal plane, recording both sections of the skiers. It was used to synchronize video and pressure insole data using dedicated software (VideoMesure, designed by the French Ski Federation), and using a right foot stomp once in the starting gate as the trigger. The video was used to identify four different consecutive phases in the turn: initiation (P1), steering1 (P2), steering2 (P3) and completion phase (P4). This turn breakdown was based on kinematic parameters and was similar to the one described by Hintermeister et al. [18]. The initiation phase was characterized by an edge change from the inside edge of the outside ski of the previous turn, to the inside edge of the novel outside ski. The steering1 phase was characterized by increased edging, until the gate passage. The steering2 phase ran from the gate passage to the maximum of hip and knee flexions. The completion phase comprised the rest of the turn. It started at the moment when skiers started to extend their lower limb and lasted until the skis were flat on the ground and the onset of the initiation phase of the following turn. The turn phases were not equal in duration. The mean initiation phase ran the first 21(±3) % of the turn duration; the steering1 phase 33 (±4)% of the turn duration; the steering2 phase 32 (± 4) % of the turn duration; and the completion phase 14 (±2)% of the turn duration.

**Fig 1:**
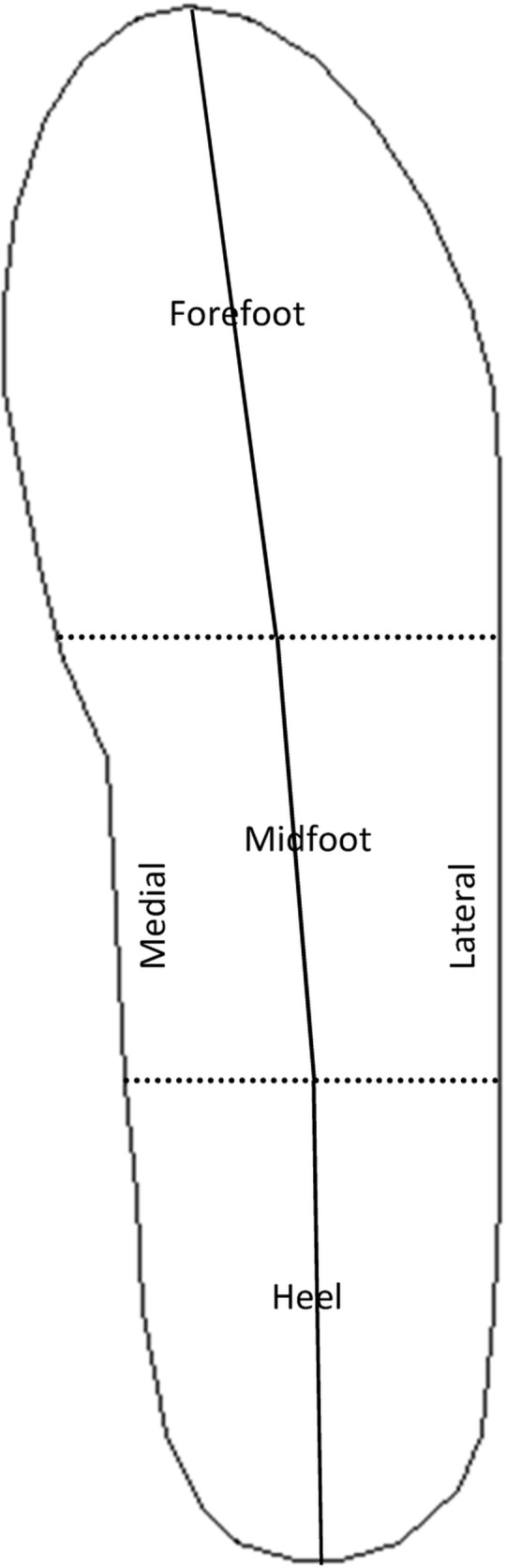
Subdivision of the plantar surface regions as used in the present study. Plantar regions distributed along the medial-lateral (separated by a solid line) and anterior-posterior (separated by a dashed line) axes are shown.

Plantar pressure was measured using a portable device (Pedar system, Novel GmbH, Munich, Germany) made up of a pair of insoles covered with 99 capacitive sensors measuring pressure under the feet’s plantar surface (kPa). The system consisted of two flexible insoles, 2 mm thick, which matched each subject’s foot size. The insoles were fitted into the ski boot’s liner. The data acquisition system was fixed on a belt that the skiers wore on their back. Before the experiment, the insoles were calibrated using the manufacturer’s guidelines. Calibrations procedure was performed while skiers were standing in the starting gate, and zeros set while lifting the corresponding foot, with buckles unhooked. Data were collected at 50 Hz throughout the entire run. Data were first saved on the system and then downloaded onto a computer after each run. The whole foot total pressure was analyzed, as was the separate pressures following the A-P and M-L axes: the foot was divided into three regions along the A-P axis (heel (H), midfoot (MF) and forefoot (FF)), and two regions along the M-L axis (medial (M) and lateral (L)). The regions taken into account in the data analysis are shown in Fig 1.

Pressure data were first multiplied by their own corresponding sensor’s area (cm^2^) to take into account size differences among the individual sensors of an insole. Thus, the resultant force acting between the plantar surface of the foot and the boot perpendicularly to the plane of the sensors’ surface (N), was obtained for each of the regions defined above, with the formula as follow:

$$F_{n}^{r_{j}}(t)=\sum_{i=1}^{n_{j}}P_{i}S_{i}$$ (1)

Where *r*_*j*_ is one of the regions, *n*_*j*_ the number of sensors of the corresponding region, *P*_*i*_ (kPa) their measured pressure, and *S*_*i*_ (m^2^) their corresponding area.

Then, the mean nGRF ($\bar{nGRF}$) was computed for the entire foot as the mean value of the sum of the force of each individual sensor. It was presented as multiples of body weight (BW). $\bar{nGRF}$ was computed on both feet, for both slope steepness and each of the four turn phases with the formula below:

$$\bar{nGRF}=\frac{\sum_{k=1}^{n}F_{n}^{r_{j}}(t)}{n}$$ (2)

Where *n* is the number of time frame for the given turn phase. This parameter represents a mean value of the force measured by the pressure insoles over a given turn phase.

The pressure time integral was calculated as the integral of pressure over time. Briefly, the force data of the sensors of a particular region were summed and then multiplied by the corresponding phase duration. Afterwards, data were divided by the entire area (cm^2^) of their particular region. For a complete description of the method used, see Melai et al. [19], and [20] for an example of how it was used. The pressure time integral was calculated for all the foot regions (total foot, H, MF, FF, M and L) and turn phases. It was expressed as a percentage of the absolute pressure time integral of a particular foot region relative to that of the entire foot (relative pressure time integral, relPTI, % of total foot PTI), for each of the four turn phases. RelPTI represents a sum of the pressure applied over an entire turn phase for a given foot region. It thus gives an indication on the relative contribution of a given foot region to the steering effect during a turn phase. Since the raw data were made of several spikes caused by the roughness of the course or natural ski vibrations, they were firstly filtered (threshold, 6 Hz) with a zero-phase low-pass fourth-order Butterworth filter [6,7].

### Statistical analysis

The means (and SD) were calculated for all variables. Data normality was checked using the Shapiro-Wilcoxon test. Several analyses of variance (ANOVA) were performed and Tukey HSD tests were used as post-hoc tests when necessary. A three-way repeated-measures ANOVA was first performed to analyze the slope effect (steep, flat), foot (inside, outside), and turn phases (P1, P2, P3, P4) on the total foot $\bar{nGRF}$. Depending on the results found in this first analysis, only the outside foot was considered in a second ANOVA, used to study the cross effects of slope and phase on the $\bar{nGRF}$ measured on the outside foot. Finally, planned comparisons were made for both M-L and A-P axes, with relPTI as the dependent variable. Turn phases P1 vs. P3 were considered, and both slopes. Statistical significance for all tests was accepted at *p*<0.05.

## Results

**Fig 2:**
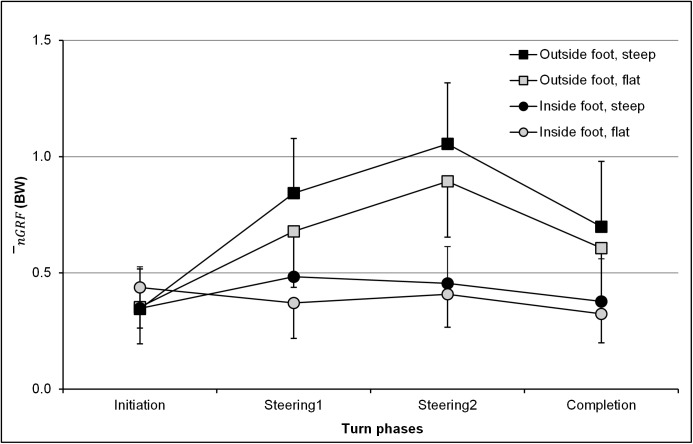
Mean normal ground reaction force ($\bar{\mathrm{nGRF}}$, in BW) under the whole foot. It is represented for both the inside (circles) and the outside (squares) feet, as well as for both the steep (full line) and flat slopes (dashed line) during the turn. Error bars represent the standard deviation of $\bar{nGRF}$.

The mean duration of turns was 1.61 (± 0.10) and 1.84 (± 0.10) s on flat and steep slopes, respectively. The mean and standard error of measurement data for the mean $\bar{nGRF}$, calculated for the four turn phases and both slopes and feet, are presented in Fig 2. Variation coefficients of less than 20% were calculated when looking at $\bar{nGRF}$ of the different turns of a same slope condition, for the same foot.

The results from the first ANOVA indicated that the mean $\bar{nGRF}$ varied significantly depending on the foot (F_*(1*,*10)*_ *= 99*.*7; p* < .001). When averaging both slopes, the outside foot was more loaded (0.68 ± 0.32 BW) than the inside foot (0.40 ± 0.17 BW). The results also suggested that the slope has a significant effect on the mean $\bar{nGRF}$ *(F*_*(1*,*10)*_ *= 7*.*0; p* < .05). When averaging both the skier’s feet, the mean $\bar{nGRF}$ was 13% higher on a steep compared to a flat slope. The turn phase also had a significant effect on the mean $\bar{nGRF}$ *(F*_*(3*,*30)*_ *= 105*.*1; p* < .001). Post-hoc tests indicated that mean $\bar{nGRF}$ applied during the initiation phase was lower (*p <* .*001) than* during the completion phase, which was itself lower (*p* < .001) than during steering1 (*p* < .001), which was also lower (*p* < .001) than during steering2.

The cross product foot × phase showed that the outside foot exhibited a significantly greater mean $\bar{nGRF}$ for the steering1 (*p* < .001), steering 2 (*p* < .001) and completion phases (*p* < .001) compared to the initiation phase (218%, 279% and 187% of the values of the mean $\bar{nGRF}$ applied during P1, respectively). For the inside foot, the mean $\bar{nGRF}$ during the completion phase was significantly lower than during both the steering1 and steering2 phases (83% (*p* < .01) and 81% (*p* < .001) of the value of the mean $\bar{nGRF}$ during steering1 and steering2, respectively). Phase-by-phase analysis indicated that the outside foot was significantly more loaded than the inside foot during turn phases P2, P3 and P4 (*p* < .001).

The cross product foot × slope showed that the outside foot was significantly affected by the slope condition (*p* < .05; +16% for the steep vs. flat slope), but not the inside foot. When analyzing both slope conditions separately, $\bar{nGRF}$ was significantly higher on the outside foot compared to the inside foot, for both the flat (*p* < .001; 0.63>0.38 BW) and steep slope conditions (*p* < .001; 0.73>0.41 BW).

To complete the analysis, a second ANOVA was performed only on the outside foot. It showed significant effects of slope *(F*_*(1*,*10)*_ *= 5*.*67; p <* .05, Steep>Flat), phase *(F*_*(3*,*30)*_ *= 94*.*81; p* < .001, P1<P4<P2<P3) and cross effect slope × phase *(F*_*(3*,*30)*_ *= 4*.*30; p* < .05) on the mean $\bar{nGRF}$. The steep slope induced significantly higher $\bar{nGRF}$ values compared to the flat slope for phases 2 and 3 (*p* < .05, Fig 2).

**Fig 3:**
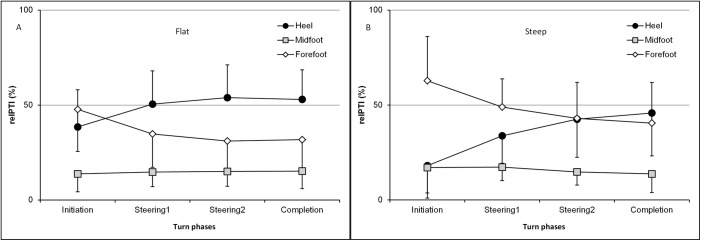
**a, b. Relative contribution of the pressure time integral (relPTI, %) applied to three plantar regions along the A-P axis.** Data of the Heel, Midfoot and Forefoot region of the outside foot are shown, for a) flat and b) steep slopes during the turn. Error bars represent the standard deviation.

**Fig 4:**
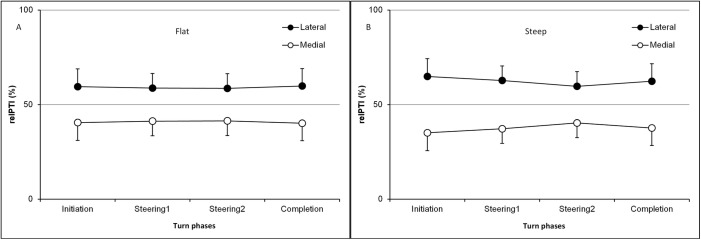
**a, b. Relative contribution of the pressure time integral (relPTI, %) applied to both the medial and lateral plantar regions.** Data are shown along the M-L axis of the outside foot, for a) flat and b) steep slopes during the turn. Error bars represent the standard deviation.

Relative loading parameter (relPTI) was used to assess the contribution of different foot sole regions, along either the M-L or the A-P axis. It was performed only on the outside foot because it was shown that $\bar{nGRF}$ applied on the inside foot i) was lower than that applied on the outside foot and ii) was not sensitive to slope steepness and turn phases. Figs 3 and 4 present the changes in relPTI along the A-P and M-L axes, respectively.

Statistical results yield a significant effect of the foot region along the anterior-posterior axis, either on flat (*F*_*(2*,*20)*_ *= 9*.*96; p* < .01) or steep slope (*F*_*(2*,*20)*_ *= 9*.*25; p* < .01). The cross product region × phase was also shown on both slope steepnesses (*F*_*(6*,*60)*_ *= 11*.*8; p* < .001 for flat slope;(*F*_*(6*,*60)*_ *= 11*.*6; p* < .001 for steep slope). No effects of the turn phase were shown. On the M-L axis, only a region effect was shown, both on the flat (*F*_*(1*,*10)*_ *= 14*.*8; p* < .01) and steep slope (*F*_*(1*,*10)*_ *= 32*.*1; p* < .001).

Further analysis was used to specifically compare regions and turn phases. Turn phases 1–3 were compared for two reasons: the initiation phase showed a different mean $\bar{nGRF}$ pattern compared to the other phases and the greatest mean $\bar{nGRF}$ was found at the end of the steering phase2. For the analysis along the A-P axis, only the FF and H regions were involved, since the MF region received only a small steady relative pressure time integral (e.g. 14.7 ±0.65% and 15.7±1.75% for the flat and steep slopes, respectively). Planned comparisons showed that relative pressure time integral on both the heel and the forefoot regions were significantly affected by turn phases, for both slope steepnesses. On the flat slope, relPTI under the FF and H regions were significantly different during P3 *(F*_*(1*,*10)*_ *= 5*.*354; p* < .05, forefoot<heel). For the steep slope, there was a significant difference in relPTI between the FF and H during P1 *(F*_*(1*,*10)*_ *= 14*.*26; p* < .01, forefoot>heel) but not during P3. Along the M-L axis, relative pressure time integral of the foot was significantly higher on the lateral half *(F*_*(1*,*10)*_ *= 26*.*360; p* < .001) for both slope conditions. No turn phase effect was shown. Detailed statistical results of the planned comparisons as well as relative pressure time integral values for certain turn phases and region are described in Table 1.

**Table 1:** Values and statistical analysis for the relative pressure time integral applied on the outside foot.

**Relative pressure time integral (%, Mean ± SD)**					
**A-P axis**			**M-L axis**		
	**Flat slope**	**Steep slope**		**Flat slope**	**Steep slope**
P1- Forefoot	47.7 ± 22.1	64.9 ± 20.3	P1- Medial	40.5 ± 9.42	35.1 ± 10.3
P1- Heel	38.5 ± 19.6	18.1 ± 17.1	P1- Lateral	59.5 ± 9.42	64.9 ± 10.3
P3- Forefoot	31.1 ± 17.5	42.7 ± 18.8	P3- medial	41.4 ± 7.78	40.3 ± 7.77
P3- Heel	53.9 ± 17.3	42.5 ± 20.1	P3- Lateral	58.6 ± 7.78	59.7 ± 7.77
**F values (planned comparisons)**					
**A-P axis**			**M-L axis**		
	**Flat slope**	**Steep slope**		**Flat slope**	**Steep slope**
Heel: P1 vs. P3	26.2***	24.4***	Medial: P1 vs. P3	0.18	2.70
Forefoot: P1 vs. P3	22.8***	18.3**	Lateral: P1 vs. P3	0.18	2.70
P1: forefoot vs. heel	0.39	14.3**	P1: medial vs. lateral	11.1**	23.1***
P3: forefoot vs. heel	5.35*	0.06	P3: medial vs. lateral	13.3**	17.0**

Data are presented as mean ± SD of the relative pressure time integral applied over a particular foot region for a given turn phase. Planned comparisons were used to analyze the cross effect of turn phases P1 and P3, either on flat or steep slope. Analysis was performed both on the A-P axis (Forefoot and Heel regions), and the M-L axis (Medial and Lateral regions). Significant differences are indicated with asterisks

(*p*<0.05:*

*p*<0.01:**

*p*<0.001:***).

## Discussion

The main findings of the present study were that i) only the $\bar{nGRF}$ under the outside foot was affected by slope steepness and turn phases, and that ii) the overall shift of a dominant loading from the forefoot toward the heel region of the outside foot was observed on both slope steepnesses, but it was posteriorly shifted on the flat slope.

### Effects of slope and turn phases on the total foot $\bar{\mathrm{nGRF}}$

**Fig 5:**
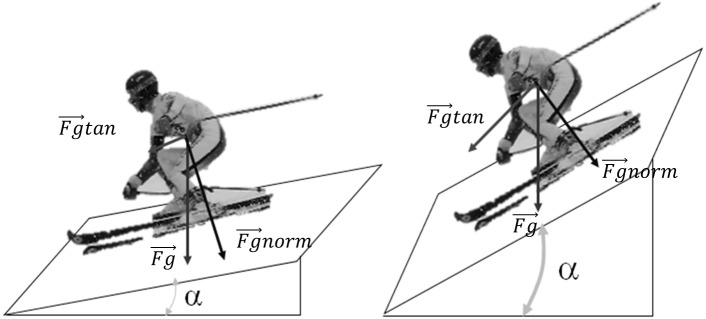
Alignment of the weight vector ($\vec{Fg}$) according to slope steepness. Its components along normal ($\vec{Fg}norm$) and tangential ($\vec{Fg}tan$) directions to the snow surface are represented. α is the slope angle.

Our results highlighted the potential effect of slope steepness on the magnitude of the force applied at the foot sole–boot interface. Skiers had to sustain higher $\bar{nGRF}$ during turning on steeper slopes. Indeed, a steeper slope increases the component of the weight vector that effectively accelerates the skier ($\vec{Fg}tan$ in Fig 5). Moreover, speed comes from a transformation of potential energy into kinetic energy in alpine skiing [12,21]. When the slope is steeper, this transformation is achieved at a higher rate, inducing a higher acceleration of the skier. Thus, in straight gliding skiing, a skier would have to control higher acceleration on steep slopes. However, a recent study actually showed that a steeper slope was correlated with a greater horizontal distance between gates in World Cup giant slalom courses [22]. This was also correlated with a slight decrease in the skier’s velocity. Another study concluded that the combination of velocity and a small turn radius leads to a high GRF that must be sustained by skiers in technical disciplines such as giant slalom [23]. In slalom skiing, it was shown that a steeper slope induced smaller turn radii and slower velocity [14]. However, to the best of our knowledge no studies have investigated the effect of slope steepness on turn radius in giant slalom. One could speculate that a slight decrease in velocity combined with a significantly reduced turn radius [23], would result in a higher centripetal force developed by skiers when turning on a steeper slope. This should be confirmed by a relatively greater lower-body lateral angulation on steeper slopes [14]. Thus, the skier’s need to control the velocity and trajectory results in a higher GRF on steeper slopes. Finally, a greater $\bar{nGRF}$ on steeper slopes would be in agreement with a higher electromyographic activity of the lower leg’s extensor muscles found on steeper slopes [24].

**Fig 6:**
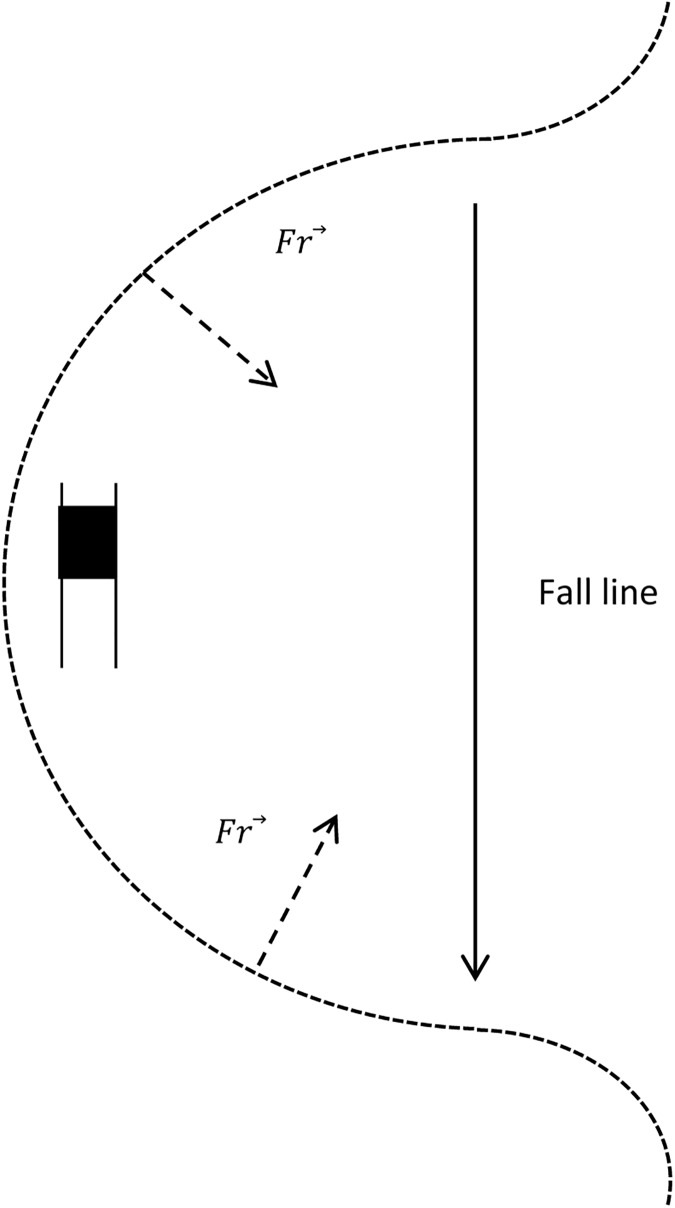
Alignment of the reaction force from snow surface (Fr⃗). Reaction force is represented at the beginning and end of a turn. The direction of the fall line is indicated.

Turn phases also played an important role in modification of $\bar{nGRF}$ in giant slalom turns. $\bar{nGRF}$ applied on the outside foot increased from the beginning of the turn to the end of the steering phase, i.e., after the gate passage [5,11,12]. However, $\bar{nGRF}$ applied under the inside foot was not sensitive to turn phases. During the steering2 phase, skiers moves away from the fall line. This may induce higher external forces that must be sustained by the skier, and this increase is managed solely by the outside foot. After gate passage, the skier’s trajectory diverges from the fall line. Hence, $\bar{nGRF}$ is opposed to the motor component of the weight. According to Newton’s action-reaction law, this might explain the increase in $\bar{nGRF}$ after gate passage (Fig 6).

Another explanation may be found in a kinematic analysis of the lower limbs. In the first part of a turn, the lower limbs flex, and then extend after the gate passage. However, lower limb flexion first decreases then increases $\bar{nGRF}$, while lower-limb extension first increases then decreases $\bar{nGRF}$ [25]. Since the shift from flexion to extension takes place during the steering2 phase of this study, this may be another explanation why the apex of the mean $\bar{nGRF}$ is encountered during this phase.

It is important to note the relative high standard deviation for the duration of the phases (e.g., relative standard deviation (RSD) 15% for P3 on flat terrain), although the duration of the turns showed a very small standard deviation (RSD 6.2% for the flat turn). This indicates a homogeneous skill level among the study’s subjects, but different strategies used for turn completion.

### Comparison of inside vs outside foot

The present study found that the effects of slope steepness and turn phases on $\bar{nGRF}$ were observable only on the outside foot. Inside and outside feet therefore have different behaviors. The present force-time series showed that a mean $\bar{nGRF}$ of the outside foot of more than 1.1 BW can be reached during a turn, whereas only 0.5 BW can be seen on the inside foot. These values were actually lower than existing data [5]. This special finding is discussed at the end of this discussion.

Statistical analysis indicated that the outside foot was more loaded than the inside foot during all conditions except the initiation turn phase. This was observed independently of the slope. This finding completes previous studies showing that $\bar{nGRF}$ is much greater for the outside than the inside foot independently of the type of turn [5] or illustrating the higher $\bar{nGRF}$ for the outside foot based on force-time series [7]. The different loading pattern for both feet induces different motor roles. As a consequence, Kröll et al. [24] noted a significantly lower activation of the vastus lateralis muscle of the inside leg.

Kinematic analysis of the lower limb could also explain part of the difference between $\bar{nGRF}$ applied to both feet. Indeed, the inside leg exhibits a significantly greater knee flexion angle as a consequence of maintaining foot–snow contact with both legs [26]. This may also be related to the force–length relationship of the quadriceps: the knee angle of the outside lower limb may place the quadriceps femoris muscle at a more suitable length to produce force compared to the inside leg [27].

As expected, $\bar{nGRF}$ was equally distributed over both the inside and outside feet during the present initiation phase of turns, i.e., when $\bar{nGRF}$ was low. This could be explained by the weight transfer from the outside ski of the previous turn to the outside ski of the current turn. Thus, skiers have already unloaded the outside ski of the former turn and progressively load the novel outside ski of the novel turn. As the turn progresses, mean $\bar{nGRF}$ applied at the outside foot increases until the end of the steering phase, i.e., after the gate passage [11,12]. In contrast, mean $\bar{nGRF}$ applied at the inside foot remained low and constant throughout the turn and was not affected by slope steepness. From a 50/50% distribution on both feet at the beginning of a turn, mean $\bar{nGRF}$ reaches up to 75%/25% distribution on the outside and inside feet, respectively, during the end of the steering phase. This ratio was not affected by slope condition.

Another interesting finding was that the influence of the slope on $\bar{nGRF}$ also depends on the foot studied. $\bar{nGRF}$ on the outside foot increased as the slope increases, whereas it remained constant on the inside foot. Thus skiers only used their outside foot to modify the mechanical parameters of a turn induced by a steeper slope.

These data highlight the different roles played by both feet during an alpine skiing giant slalom turn. Both feet are responsible for the skier’s stability, although only the outside foot has an active motor role in turn completion. In order to turn, skiers need to edge and bend their skis, which then creates centripetal force. To maintain their stability, the skier needs to control external forces and torques applied during turning. A higher $\bar{nGRF}$ on the outside foot may be needed to control the reaction force’s alignment with the skier’s center of gravity, hence allowing the skier to stay in balance while turning. Indeed, skiers would have to lean too far toward the inside of the turn if a large $\bar{nGRF}$ is applied on the inside foot. Moreover, and for reasons already explained, skiers would be unable to sustain high torques generated by external forces on the inside leg.

In competitive alpine skiing, gate settings induce a turn radius that is actually tighter than the ski radius, especially on steep slope [22]. Hence, skiers have to push on their skis to bend them, in order to make them turn along a tighter turn radius [11]. Turn radii of the outside ski as low as 12 m have been measured in World Cup giant slalom ski racing [21]. Such small turn radii may not be reached if skiers exert an even force on both skis. Hence, strong pressure on one ski may be needed to effectively perform a carving turn with a small turn radius. For stability purposes explained above, that strong pressure can only be realized on the outside foot.

### Relative loading distribution along the anterior-posterior axis of the outside foot

Relative loading on the A-P axis was significantly affected by the foot region. A shift was observed in the dominant foot region from the forefoot to the heel during a turn. These results indicated that $\bar{nGRF}$ acts predominantly through the forefoot during the turn initiation. This relates to a body leaning forward with the shin pressing on the front part of the boot [10], necessary for proper bending of the ski [28]. This must also be related to a CoP moving forward on the skis and boot [8].

The present study has indicated that the inclination of the slope does not influence this A-P shift during the turn: heel relative loading increases during the turn until it equals (steep slope) or exceeds (flat slope condition) the forefoot relative loading (Fig 3).

However, the distribution between the heel and the forefoot within each turn phase was significantly modified by both the inclination of the slope and the turn phases. On the steeper slope, the relative loading was i) greater for the forefoot compared to the heel during the initiation phase and ii) similar for both forefoot and heel regions during the steering phase. On the contrary, on a flatter slope, relative loading was i) similar for both the forefoot and heel regions during the initiation phase and ii) greater under the heel than under the forefoot region at the end of the steering phase. When the slope was steep, skiers needed to increase the $\bar{nGRF}$ on the anterior part of the ski at the beginning of the turn to initiate the turn quickly and therefore transfer their body weight forward. In contrast, when the slope was low, the mechanical constraints differed and skiers tended to remain centered in their boots. They were more focused on optimizing gliding properties by avoiding overpressures (Fig 3).

There is a forward shift of the skier’s body weight when the slope steepness increased. Indeed, the weight vector crosses the plane formed by both skis more anteriorly, as is described in Fig 5. These results also suggest that the range of motion along the A-P axis is greater on steeper slopes. Although no statistic is available, this is confirmed by Nakazato et al. who measured a range of motion of the CoP along the A-P axis of 271.7 mm on a steep slope and 195.5 mm on flat slope [8]. A greater range of motion of the CoP along the A-P axis on a steep slope indicates a greater need to manage the skier’s balance.

A relation between a steeper slope, a shorter turn radius and a greater $\bar{nGRF}$ could also explain the higher forefoot relative loading during turn initiation on the steep slope. This force is better created when leaning forward [15]. Moreover, it has been shown that higher ranked skiers are better able to produce a greater loading under the front part of the outside foot [13].

In contrast, relative loading applied through the midfoot region remained low and stable. Furthermore, it was not affected by modifications of slope steepness or turn phases. Low and steady pressures were applied through the midfoot region, and the foot’s arch prevents the foot from transferring large forces through that region.

### Relative loading distribution along the medial-lateral axis of the outside foot

These results showed that the relative loading was always applied about 40% and 60% on the medial and lateral halves of the outside foot, respectively. This agreed with earlier results of pressure insole measurement, which showed that CoP was always lateral on the outside foot [8]. Indeed, rigid ski boots have only one degree of freedom along the plantar flexion–dorsiflexion axis, thus limiting athletes from effectively acting on the M-L loading distribution [29]. M-L pressure distribution may differ if measured under the plantar surface of the foot or at the shin–boot interface, which was not the case in this study [10,30].

Although this was not significant, the results reported herein showed that a steep slope induced an even greater relative loading applied on the lateral half of the plantar surface. This M-L distribution was not affected by turn phase or slope during skiing.

However, GRF from the snow acts on the inside edge of the skis, as shown by the literature and experimental observations [16,31]. Therefore, it was expected that i) the medial half of the outside foot was more loaded than the lateral half and that ii) the lateral half of the inside foot was more loaded than the medial half. This hypothesis was based on the study of Federolf et al. [16]. They found that, when edging the skis in straight gliding-test skiing (i.e., the distance between the knees is smaller than that between the ankles, as seen in the frontal plane), the force application point along the M-L axis was shifted toward the inside edge of the skis. Secondly, the contact point between the snow and the ski occurred at the inside edge of the ski. The contact point between the plantar surface of the foot and the boot was spread over a distance that was longer than the ski’s width [31]. Finally, the moment arm along the longitudinal axis of the ski when performing a turn (Mx, as described in ISO 9462), tended to approach the contact point at the foot–boot interface and at the snow–ski interface. However, the data presented in Niessen et al. and Federolf et al. were obtained with a force platform placed under the boot or the bindings, respectively [16,31]. It has been shown that the CoP lies medially on the outside foot and laterally on the inside foot during a turn, when measured using a force platform [8,32]. Hence, pressure insoles and force platforms provide truly distinct information regarding the skier’s kinetics. Pressure insoles provide valuable information on the kinaesthetic feeling of the skier while performing turns. Depending on their design, force platforms provide from one to six components of the forces and moments acting between the snow and the skier. In this aspect, they can be considered as a true measurement of GRF acting on the skier.

### Limitations of the study

A number of limitations of the present study should be noted. Firstly, pressure insoles are a useful tool to measure GRF based on raw plantar pressure measurements. However, it has been shown GRF values measured with pressure insoles tended to be underestimated when compared to measurements made with force platforms [6,7,33]. This is thought to be due to rigid ski boots going up high on the shank. This tended to be confirmed by GRF measurements that were shown to be more reliable in other activities with low-cut shoes, such as walking [34,35]. Indeed, some force between the foot and the ski boot is actually exerted through the ski boot cuff [10]. Hence, plantar pressure measurements may not reflect the exact GRF value. Moreover, this amount of force not measured by pressure insoles may actually depend on the ankle flexion angle: the underestimation of GRF with pressure insoles may vary during a turn.

Secondly, we measured quite low nGRF values, even when compared to other studies which used pressure insoles [5]. This could be explained by several factors. The subjects were not top-level ski racers, although they were very good ski racers and regularly competed in FIS competitions. It has been shown that higher-ranked skiers exert greater GRF [13]. Moreover, pressure data were smoothed using a filter with a cut off frequency at 6 Hz. Last but not least, force and pressure data were presented as means over an entire turn phase, which automatically lowers values, whereas other articles usually presented peak values [5,7].

## Conclusion

Pressure insoles were chosen because they can distinguish between pressures applied at different regions under the sole of the foot, and therefore determine how the force applied normally to their surface during a turn evolves. This descriptive article highlighted the different roles played by both feet during alpine skiing, according to the phase of the turn and the slope steepness. Mean nGRF under the inside foot remained low and was not modified by changes in the turn phases or slope steepness. In contrast, the mean nGRF applied under the outside foot was higher except during initiation phases. Moreover, it intensified i) when slope steepness increased and ii) from the beginning of a turn to the end of the steering phase. Hence, the outside foot took an active part in the turning process, while the inside foot had more of a supporting role. Relative loading distribution of the outside foot along the ski axis was also affected by turn characteristics. There was a forward-oriented shift of the skier’s body weight i) at the beginning of turns and ii) this was more pronounced for steeper slopes. On the other hand, the M-L distribution of the relative loading was only slightly influenced by both slope and turn phases, and remained always dominant on the lateral half of the foot. The present results may be of particular interest for athletes, coaches and boot manufacturers.

## Supporting information

S1 TableDetailed results of $\bar{\mathrm{nGRF}}$ (in multiples of BW) applied under the entire plantar surface.Results are classified according to the foot position, the slope steepness and the turn phases.(PDF)

S2 Table Detailed results of the relative pressure time integral (%) applied under the outside foot. Results are classified according to the slope steepness (flat and steep), turn phases (P1 to P4) and foot regions (Heel, Midfoot and forefoot).
(PDF)

S3 Table Detailed results of the relative pressure time integral (%) applied under the outside foot. Results are classified according to the slope steepness (flat and steep), turn phases (P1 to P4) and foot regions (Medial and lateral).
(PDF)

## References

[1] Girard O, Eicher F, Fourchet F, Micallef JP, Millet GP. Effects of the playing surface on plantar pressures and potential injuries in tennis. Br J Sports Med. 2007;41: 733–738. doi: 10.1136/bjsm.2007.036707 17566048 10.1136/bjsm.2007.036707 PMC2465293

[2] Stöggl T, Müller E, Lindinger S. Biomechanical comparison of the double-push technique and the conventional skate skiing technique in cross-country sprint skiing. J Sports Sci. 2008;26: 1225–1233. doi: 10.1080/02640410802027386 18720201 10.1080/02640410802027386

[3] Lafontaine D, Lamontagne M, Dupuis D, Diallo B. Plantar pressure distribution measured during alpine ski turns. VI Emed Scientific Meeting,. Brisbane, Australia.; 1998.

[4] Lafontaine D, Lamontagne M, Dupuis D, Diallo B. Analysis of the distribution of pressure under the feet of elite alpine ski instructors. XVI International Symposium on Biomechanics in Sports. Konstanz, Germany.; 1998.

[5] Lamontagne M. Plantar pressure distribution and forces measured during slalom and giant slalom turns performed by elite skiers. 19th International Symposium on Biomechanics in Sports. San Francisco; 2001. pp. 211–214.

[6] Stricker G, Scheiber P, Lindenhofer E, Müller E. Determination of forces in alpine skiing and snowboarding: Validation of a mobile data acquisition system. Eur J Sport Sci. 2010;10: 31–41.

[7] Nakazato K, Scheiber P, Müller E. A comparison of ground reaction forces determined by portable force-plate and pressure-insole systems in alpine skiing. J Sport Sci Med. 2011/01/01. 2011;10: 754–762. Available: http://www.ncbi.nlm.nih.gov/pubmed/24149570 PMC3761512 24149570

[8] Nakazato K, Scheiber P, Müller E. Comparison between the force application point determined by portable force plate system and the center of pressure determined by pressure insole system during alpine skiing. Sport Eng. 2013;16: 297–307.

[9] Scheiber P, Schwameder H, Müller E. Characteristics of the force application point—a method to identify learning processes in alpine skiing? In: Schwameder H, Strutzenberger G, Fastenbauer V, Lindinger S, Müller E, editors. XXIV international symposium on biomechanics in sports. Salzburg; 2006. pp. 564–567.

[10] Kersting UG, Kurpiers N, Hild E, Kiefmann A, Senner V. Comparison of a six degree-of-freedom force sensor and pressure insole measurements in selected skiing manoeuvres. Emed Scientific Meeting, ESM. Providence, RI, USA.; 2010. p. 55.

[11] Müller E, Schwameder H. Biomechanical aspects of new techniques in alpine skiing and ski-jumping. J Sports Sci. 2003;21: 679–692. doi: 10.1080/0264041031000140284 14579866 10.1080/0264041031000140284

[12] Meyer F. Biomechanical analysis of alpine skiers performing giant slalom turns. Université de Lausanne 2011.

[13] Keränen T, Ihalainen S, hynynen E, Salo T. FIS-ranking and carving turn force production profile. 5th International Congress on Science and Skiing. Salzburg; 2010.

[14] Supej M, Hebert-Losier K, Holmberg HC. Impact of the steepness of the slope on the biomechanics of World Cup slalom skiers. Int J Sport Physiol Perform. 2015;10: 361–368. 10.1123/ijspp.2014-0200 25229249

[15] Reid R. A kinematic and kinetic study of alpine skiing technique in slalom. Norwegian School of Sport Science 2010.

[16] Federolf P, Scheiber P, Rauscher E, Schwameder H, Lüthi A, Rhyner HU, et al Impact of skier actions on the gliding times in alpine skiing. Scand J Med Sci Sports. 2008;18: 790–797. doi: 10.1111/j.1600-0838.2007.00745.x 18248548 10.1111/j.1600-0838.2007.00745.x

[17] Gilgien M, Spörri J, Chardonnens J, Kröll J, Müller E. Determination of External Forces in Alpine Skiing Using a Differential Global Navigation Satellite System. Sensors. 2013;13: 9821–9835. doi: 10.3390/s130809821 23917257 10.3390/s130809821 PMC3812581

[18] Hintermeister RA, O’Connor DD, Dillman CJ, Suplizio CL, Lange GW, Steadman JR. Muscle activity in slalom and giant slalom skiing. Med Sci Sport Exerc. 1995;27: 315–322. 7752856

[19] Melai T, Ijzerman TH, Schaper NC, de Lange TLH, Willems PJB, Meijer K, et al Calculation of plantar pressure time integral, an alternative approach. Gait Posture. 2011;34: 379–383. doi: 10.1016/j.gaitpost.2011.06.005 21737281 10.1016/j.gaitpost.2011.06.005

[20] Fourchet F, Kelly L, Horobeanu C, Loepelt H, Taiar R, Millet GP. Comparison of plantar pressure distribution in adolescent runners at low vs. high running velocity. Gait Posture. 2012;35: 685–687. doi: 10.1016/j.gaitpost.2011.12.004 22205042 10.1016/j.gaitpost.2011.12.004

[21] Supej M. Differential specific mechanical energy as a quality parameter in racing alpine skiing. J Appl Biomech. 2008;24: 121–129. 18579904 10.1123/jab.24.2.121

[22] Gilgien M, Crivelli P, Sporri J, Kroll J, Muller E. Characterization of course and terrain and their effect on skier speed in world cup alpine ski racing. PLoS One. 2015;10. 10.1371/journal.pone.0118119 PMC4356573 25760039

[23] Gilgien M, Sporri J, Kroll J, Crivelli P, Muller E. Mechanics of turning and jumping and skier speed are associated with injury risk in men’s World Cup alpine skiing: a comparison between the competition disciplines. Br J Sport Med. 2014;48: 742–747. 10.1136/bjsports-2013-092994 24489379

[24] Kroll J, Wakeling JM, Seifert JG, Muller E. Quadriceps Muscle Function during Recreational Alpine Skiing. Med Sci Sport Exerc. 2010;42: 1545–1556. 10.1249/MSS.0b013e3181d299cf 20068490

[25] Bentley JR, Amonette WE, De Witt JK, Hagan RD. Effects of different lifting cadences on ground reaction forces during the squat exercise. J Strength Cond Res. 2010;24: 1414–1420. doi: 10.1519/JSC.0b013e3181cb27e7 20386484 10.1519/JSC.0b013e3181cb27e7

[26] Kröll J, Spörri J, Kandler C, Fasel B, Müller M, Schwameder H. Kinetic and kinematic comparison of alpine ski racing disciplines as a base for specific conditioning regimes. 33rd International society of biomechanics in sports. Poitiers, France; 2015.

[27] Marginson V, Eston R. The relationship between torque and joint angle during knee extension in boys and men. J Sport Sci. 2001/11/07. 2001;19: 875–880. 10.1080/026404101753113822 11695509

[28] Yoneyama T, Kitade M, Osada K. Investigation on the ski-snow interaction in a carved turn based on the actual measurement. Procedia Eng. 2010;2: 2901–2906.

[29] Petrone N, Marcolin G, Panizzolo FA. The effect of boot stiffness on field and laboratory flexural behavior of alpine ski boots. Sport Eng. 2013;16: 265–280.

[30] Schaff P, Senner V, Kaiser F. Pressure distribution measurements for the alpine skier—from the biomechanical high tech measurement to its application as Swingbeep-feedback system In: Mueller E, editor. Skiing and Science. London, E & FN Spon; 1997 pp. 159–172.

[31] Niessen W, Müller E, Schwameder H, Wimmer MA, Riepler B. Force and moment measurements during alpine skiing depending on height position. XXVI International Symposium on Biomechanics in Sports. Konstanz, Germany; 1999.

[32] Senner V, Mitternacht J, Hermann A, Rauner K, Supej M. The point of force application during the turn and its meaning for science in skiing. 2016.

[33] Lüthi A, Federolf P, Fauve M, Oberhofer K, Rhyner HU, Ammann W, et al Determination of forces in carving using three independant methods In: Müller D. Klika R. Lindinger S Schwameder H. EB, editor. Science and Skiing. Oxford: Meyer & Meyer Sport Ltd; 2005 pp. 96–106.

[34] Chesnin KJ, Selby-Silverstein L, Besser MP. Comparison of an in-shoe pressure measurement device to a force plate, concurrent validity of center of pressure measurements. Gait Posture. 2000;12: 128–133. 10998609 10.1016/s0966-6362(00)00071-0

[35] Forner Cordero A, Koopman HJFM, van der Helm FCT. Use of pressure insoles to calculate the complete ground reaction forces. J Biomech. 2004;37: 1427–1432. doi: 10.1016/j.jbiomech.2003.12.016 15275851 10.1016/j.jbiomech.2003.12.016

