# Supplementary material for: Influence of slope steepness, foot position and turn phase on plantar pressure distribution during giant slalom alpine ski racing
Source: PLoS One. 2017 May 4;12(5):e0176975. doi: 10.1371/journal.pone.0176975 (PMC5417654; doi:10.1371/journal.pone.0176975)
Supplement: S1 Table — Results are classified according to the foot position, the slope steepness and the turn phases. (PDF) [file pone.0176975.s001.pdf]

	Mean vGRF (N/BW) on the entire plantar surface															
	Inside foot								Outside foot							
	Flat				Steep				Flat				Steep			
Subjects	P1	P2	P3	P4	P1	P2	P3	P4	P1	P2	P3	P4	P1	P2	P3	P4
1	0,65	0,40	0,41	0,38	0,28	0,46	0,52	0,39	0,23	0,75	1,13	0,74	0,35	0,78	1,26	1,13
2	0,25	0,18	0,23	0,15	0,10	0,29	0,26	0,15	0,25	0,52	0,65	0,34	0,10	0,57	0,70	0,28
3	0,31	0,28	0,44	0,21	0,20	0,43	0,44	0,26	0,26	0,50	0,67	0,41	0,23	0,63	0,65	0,37
4	0,41	0,47	0,50	0,35	0,36	0,47	0,45	0,32	0,42	0,79	0,98	0,73	0,32	0,70	1,05	0,47
5	0,41	0,41	0,42	0,46	0,42	0,44	0,42	0,30	0,34	0,59	0,84	0,62	0,32	0,94	1,02	0,66
6	0,06	0,03	0,11	0,08	0,04	0,12	0,14	0,11	0,09	0,25	0,39	0,15	0,17	0,69	0,99	0,45
7	0,53	0,40	0,51	0,33	0,40	0,60	0,41	0,45	0,46	0,66	0,96	0,76	0,51	0,98	1,13	0,76
8	0,50	0,45	0,38	0,37	0,35	0,62	0,55	0,38	0,32	0,66	0,95	0,75	0,33	0,70	0,83	0,85
9	0,50	0,44	0,33	0,37	0,55	0,54	0,53	0,53	0,37	0,74	1,06	0,74	0,33	0,80	1,08	0,72
10	0,58	0,48	0,57	0,47	0,50	0,83	0,52	0,49	0,70	1,23	1,21	0,91	0,75	1,32	1,45	0,95
11	0,60	0,56	0,58	0,37	0,61	0,52	0,75	0,76	0,44	0,76	1,00	0,51	0,38	1,16	1,44	1,04
Mean	0,44	0,37	0,41	0,32	0,35	0,48	0,45	0,38	0,35	0,68	0,89	0,61	0,34	0,84	1,05	0,70
SD	0,18	0,15	0,14	0,12	0,18	0,18	0,16	0,18	0,16	0,24	0,24	0,23	0,17	0,23	0,26	0,28
