# Supplementary material for: Influence of slope steepness, foot position and turn phase on plantar pressure distribution during giant slalom alpine ski racing
Source: PLoS One. 2017 May 4;12(5):e0176975. doi: 10.1371/journal.pone.0176975 (PMC5417654; doi:10.1371/journal.pone.0176975)
Supplement: S2 Table — Results are classified according to the slope steepness (flat and steep), turn phases (P1 to P4) and foot regions (Heel, Midfoot and forefoot). (PDF) [file pone.0176975.s002.pdf]

**Rel PTI (%) on the Anterior-posterior axis**

| | Outside foot | | | | | | | | | | | | | | | | | | | | | | | |
|---|---|---|---|---|---|---|---|---|---|---|---|---|---|---|---|---|---|---|---|---|---|---|---|---|
| | **Flat** | | | | | | | | | | | | **Steep** | | | | | | | | | | | |
| | P1 | | | P2 | | | P3 | | | P4 | | | P1 | | | P2 | | | P3 | | | P4 | | |
| Subjects | Heel | Midfoot | Forefoot | Heel | Midfoot | Forefoot | Heel | Midfoot | Forefoot | Heel | Midfoot | Forefoot | Heel | Midfoot | Forefoot | Heel | Midfoot | Forefoot | Heel | Midfoot | Forefoot | Heel | Midfoot | Forefoot |
| 1 | 32,94 | 8,08 | 58,98 | 64,38 | 11,25 | 24,37 | 72,43 | 11,56 | 16,01 | 80,15 | 5,51 | 14,34 | 16,70 | 12,59 | 70,71 | 29,22 | 20,55 | 50,23 | 65,76 | 9,34 | 24,90 | 67,32 | 3,95 | 28,74 |
| 2 | 41,57 | 26,70 | 31,74 | 52,68 | 27,40 | 19,92 | 56,22 | 26,26 | 17,52 | 55,83 | 25,87 | 18,30 | 55,20 | 19,63 | 25,17 | 34,68 | 23,21 | 42,11 | 44,51 | 22,53 | 32,96 | 33,37 | 33,75 | 32,89 |
| 3 | 30,66 | 4,78 | 64,56 | 51,90 | 8,96 | 39,14 | 50,17 | 10,20 | 39,63 | 41,00 | 6,66 | 52,34 | 14,52 | 4,77 | 80,72 | 32,50 | 12,15 | 55,35 | 35,38 | 14,28 | 50,34 | 52,52 | 8,69 | 38,79 |
| 4 | 37,82 | 6,13 | 56,05 | 45,62 | 12,67 | 41,70 | 46,02 | 11,02 | 42,96 | 41,14 | 9,65 | 49,21 | 24,87 | 5,25 | 69,88 | 34,17 | 9,23 | 56,60 | 47,65 | 8,90 | 43,45 | 50,59 | 6,39 | 43,03 |
| 5 | 33,69 | 3,15 | 63,16 | 44,73 | 2,81 | 52,46 | 42,17 | 6,55 | 51,29 | 55,85 | 2,24 | 41,92 | 5,59 | 9,22 | 85,19 | 33,06 | 9,24 | 57,69 | 40,97 | 7,78 | 51,25 | 31,53 | 3,65 | 64,83 |
| 6 | 59,80 | 21,02 | 19,18 | 69,00 | 17,33 | 13,66 | 67,84 | 21,43 | 10,73 | 55,49 | 36,24 | 8,26 | 0,04 | 16,10 | 83,86 | 22,47 | 24,66 | 52,87 | 14,22 | 22,21 | 63,57 | 6,82 | 20,69 | 72,48 |
| 7 | 12,87 | 7,27 | 79,86 | 23,97 | 10,70 | 65,33 | 31,40 | 11,87 | 56,74 | 32,21 | 10,27 | 57,52 | 13,35 | 5,58 | 81,07 | 12,56 | 10,06 | 77,38 | 10,69 | 8,38 | 80,93 | 21,63 | 11,45 | 66,93 |
| 8 | 37,06 | 17,71 | 45,23 | 48,40 | 15,08 | 36,52 | 59,20 | 16,09 | 24,70 | 59,25 | 15,20 | 25,55 | 4,67 | 38,91 | 56,42 | 33,55 | 25,02 | 41,44 | 46,96 | 22,37 | 30,67 | 62,57 | 19,38 | 18,04 |
| 9 | 36,38 | 33,44 | 30,18 | 44,24 | 24,51 | 31,25 | 50,49 | 21,42 | 28,09 | 45,06 | 27,04 | 27,90 | 16,00 | 44,74 | 39,26 | 41,20 | 24,77 | 34,03 | 45,16 | 20,52 | 34,31 | 56,38 | 24,63 | 18,99 |
| 10 | 16,38 | 17,38 | 66,24 | 26,21 | 23,22 | 50,56 | 29,66 | 22,28 | 48,06 | 38,48 | 19,12 | 42,40 | 4,82 | 19,51 | 75,67 | 27,06 | 21,71 | 51,23 | 34,93 | 20,53 | 44,54 | 34,99 | 13,71 | 51,30 |
| 11 | 84,22 | 5,79 | 9,99 | 84,21 | 8,08 | 7,72 | 87,17 | 6,70 | 6,12 | 78,22 | 9,73 | 12,05 | 42,82 | 11,48 | 45,70 | 71,10 | 9,58 | 19,33 | 81,42 | 5,46 | 13,13 | 85,98 | 4,70 | 9,32 |
| Mean | 38,49 | 13,77 | 47,74 | 50,49 | 14,73 | 34,78 | 53,89 | 15,04 | 31,08 | 52,97 | 15,23 | 31,80 | 18,05 | 17,07 | 64,88 | 33,78 | 17,29 | 48,93 | 42,51 | 14,75 | 42,73 | 45,79 | 13,72 | 40,48 |
| SD | 19,55 | 10,13 | 22,14 | 17,53 | 7,66 | 17,56 | 17,31 | 6,82 | 17,51 | 15,57 | 10,64 | 17,57 | 17,07 | 13,39 | 20,33 | 14,47 | 7,09 | 14,86 | 20,09 | 6,93 | 18,80 | 22,62 | 9,81 | 21,39 |
