# Supplementary material for: Influence of slope steepness, foot position and turn phase on plantar pressure distribution during giant slalom alpine ski racing
Source: PLoS One. 2017 May 4;12(5):e0176975. doi: 10.1371/journal.pone.0176975 (PMC5417654; doi:10.1371/journal.pone.0176975)
Supplement: S3 Table — Results are classified according to the slope steepness (flat and steep), turn phases (P1 to P4) and foot regions (Medial and lateral). (PDF) [file pone.0176975.s003.pdf]

Rel PTI (%) on the Medial-lateral axis

	Outside foot															
	**Flat**								**Steep**							
	P1		P2		P3		P4		P1		P2		P3		P4	
Subjects	Medial	Lateral	Medial	Lateral	Medial	Lateral	Medial	Lateral	Medial	Lateral	Medial	Lateral	Medial	Lateral	Medial	Lateral
1	56,36	43,64	44,31	55,69	41,23	58,77	39,68	60,32	40,15	59,85	34,17	65,83	37,73	62,27	42,84	57,16
2	40,59	59,41	49,92	50,08	52,06	47,94	47,38	52,62	16,62	83,38	36,53	63,47	41,78	58,22	29,83	70,17
3	43,08	56,92	38,70	61,30	41,93	58,07	43,45	56,55	44,44	55,56	39,35	60,65	43,93	56,07	41,32	58,68
4	47,60	52,40	46,23	53,77	48,69	51,31	50,46	49,54	50,09	49,91	48,71	51,29	51,61	48,39	47,19	52,81
5	52,61	47,39	52,94	47,06	48,87	51,13	51,39	48,61	35,30	64,70	38,60	61,40	42,80	57,20	48,15	51,85
6	30,42	69,58	32,90	67,10	28,70	71,30	21,43	78,57	37,18	62,82	24,56	75,44	24,57	75,43	17,27	82,73
7	42,05	57,95	40,24	59,76	43,33	56,67	42,44	57,56	45,75	54,25	45,45	54,55	48,59	51,41	46,41	53,59
8	29,96	70,04	39,07	60,93	40,09	59,91	40,24	59,76	27,72	72,28	38,42	61,58	39,43	60,57	39,25	60,75
9	37,16	62,84	43,52	56,48	43,48	56,52	41,53	58,47	25,54	74,46	38,97	61,03	44,91	55,09	36,29	63,71
10	39,81	60,19	41,05	58,95	40,58	59,42	37,74	62,26	38,46	61,54	37,64	62,36	38,25	61,75	36,03	63,97
11	26,15	73,85	25,09	74,91	26,88	73,12	26,15	73,85	25,16	74,84	27,31	72,69	30,06	69,94	29,70	70,30
Mean	40,52	59,48	41,27	58,73	41,44	58,56	40,17	59,83	35,13	64,87	37,25	62,75	40,33	59,67	37,66	62,34
SD	9,42	9,42	7,71	7,71	7,78	7,78	9,25	9,25	10,26	10,26	6,92	6,92	7,77	7,77	9,30	9,30
